# The Caries Management System: are preventive effects sustained postclinical trial?

**DOI:** 10.1111/cdoe.12204

**Published:** 2015-12-07

**Authors:** R. Wendell Evans, Paula Clark, Nan Jia

**Affiliations:** ^1^ Population Oral HealthSydney Dental SchoolUniversity of SydneySydneyNSWAustralia; ^2^ Lilly Corporate CenterEli Lilly and CompanyIndianapolisINUSA

**Keywords:** cariology, dental services research, non‐surgical treatment, preventive dentistry, risk assessment

## Abstract

**Objectives:**

To report, at two and 4 years post‐trial, on the potential legacy of a 3‐year randomized controlled clinical trial (RCT) of the Caries Management System (CMS) at private general dental practices. The CMS was designed to reduce caries risk and need for restorative care.

**Methods:**

Nineteen dental practices located in city, urban, and rural locations in both fluoridated and nonfluoridated communities participated in the RCT. Eight practices were lost to follow‐up post‐trial; however, baseline mean DMFT balance between CMS and control practices was maintained. At the control practices, caries management following usual practice continued to be delivered. The patient outcome measure was the cumulative increment in the DMFT index score, and the practice outcome measures included the practice‐mean and practice‐median increments of patient DMFT index scores. In covariable analysis (patient‐level unit of analysis), as the patients were clustered by practices, mean DMFT increments were determined through multilevel modeling analysis. Practice‐mean DMFT increments (practice‐level unit of analysis) and practice‐median DMFT increments (also practice level) were determined through general linear modeling analysis of covariance. In addition, a multiple variable logistic regression analysis of caries risk status was conducted.

**Results:**

The overall 4‐year post‐trial result (years 4–7) for CMS patients was a DMFT increment of 2.44 compared with 3.39 for control patients (*P* < 0.01), a difference equivalent to 28%. From the clinical trial baseline to the end of the post‐trial follow‐up period, the CMS and control increments were 6.13 and 8.66, respectively, a difference of 29% (*P* < 0.0001). Over the post‐trial period, the CMS and control practice‐mean DMFT increments were 2.16 and 3.10 (*P* = 0.055) and the respective increments from baseline to year 7 were 4.38 and 6.55 (*P* = 0.029), difference of 33%. The practice‐median DMFT increments during the 4‐year post‐trial period for CMS and control practices were 1.25 and 2.36 (*P* = 0.039), and the respective increments during the period from baseline to year 7 were 2.87 and 5.36 (*P* < 0.01), difference of 47%. Minimally elevated odds of being high risk were associated with baseline DMFT (OR = 1.17). Patients attending the CMS practices had lower odds of being high risk than those attending control practices (OR = 0.23, 95% CI = 0.06, 0.88).

**Conclusion:**

In practices where adherence to the CMS protocols was maintained during the 4‐year post‐trial follow‐up period, patients continued to benefit from a reduced risk of caries and, therefore, experienced lower needs for restorative treatment.

The landmark longitudinal study of Backer Dirks demonstrated that dental caries is a dynamic disease characterized by varying rates of progression, arrest, and remineralization 1. This should have pointed dentistry toward a more conservative approach to caries management. The longitudinal study of Rugg‐Gunn indicated that over‐treatment of caries could be reduced if the threshold for surgical intervention was set at the enamel cavitation stage, yet the radical surgical approach prevailing in the 1960s has generally continued despite overwhelming research outputs in caries prevention and treatment in the succeeding half century 2, 3. Recently, Baelum has pleaded for a biological approach to caries treatment, one that addresses causative factors and their control rather than the mechanical approach of merely eradicating individual lesions as they arise 4.

## The Caries Management System

The Caries Management System (CMS) was inspired by Axelsson et al. 5, 6 who demonstrated that caries incidence in children and young and older adults could be reduced to near zero levels and that such outcome could be sustained for decades. We realized that dental practices did not adequately and comprehensively address caries prevention. The CMS protocols were designed to deliver two clinical outcomes: to prevent caries incidence and to arrest existing noncavitated lesions thus preventing their progression to cavities and consequent need for restorative treatment. The guiding treatment philosophy is that noncavitated lesions should not be restored, rather they should be actively arrested, remineralized, and monitored.

The CMS comprises a set of protocols (covering risk assessment, diagnosis, risk management, monitoring, and recall) that bring together evidence‐based caries preventive methods in a systematic framework 7, 8. It specifies how they should be delivered to patients who are at different levels of caries risk. Treatment set out in the protocols is risk‐specific; therefore, each patient's caries risk must be determined at the outset. Briefly, for new patients, those at high risk present with cavities; medium‐risk patients present with teeth exhibiting signs of enamel breakdown (ICDAS stage 3 lesions) or approximal lesions as shown in bitewing radiographs where lesion depth is confined to the outer third of dentine; and low‐risk patients are those who present with radiolucencies of lesser depth or with clinical signs of not greater than ICDAS stage 2 lesions. Thereafter, patients are rated as high risk if they develop more than one new caries lesion per year; medium‐risk patients develop one new lesion per year, or have progression of existing lesions; and low‐risk patients are those with lesion incidence of fewer than one per year.

The CMS focus is on the management of patient behavior change (oral hygiene coaching, selection of healthy diet components, and encouragement to restrict between‐meal exposures to sugar‐containing foods and beverages) and the nonsurgical clinical treatment of noncavitated lesions. The nonsurgical clinical care entails fluoride varnish applications to prevalent noncavitated lesions, the frequency of which is risk‐determined; 3‐monthly applications for high‐risk patients and 6 monthly for medium risk 7, 9. Sealants are used both preventively and therapeutically 10. Caries risk reduction is monitored on the basis of lesion activity, that is, from direct clinical and radiographic observations on lesion incidence and progression. Surgical treatment of cavities, in terms of caries control, does not control the disease but does eliminate lesions and sites for plaque buildup 11.

## The monitor practice program

The Monitor Practice Program (MPP) began as a multicenter cluster randomized controlled clinical trial designed to test the hypothesis that the evidence‐based CMS protocols, tested previously in a hospital setting, would reduce risk of dental caries experience in patients attending privately operated general dental practices (Clinical Trial Registry No. ISRECTN67374556) 12, 13.

The MPP was planned and implemented initially as a 3‐year trial, and during this period, investigator contacts occurred frequently to monitor the transition to the CMS protocols and to monitor outcomes 13, 14. At year 3, by intention to treat, caries incidence (the D component of the DMFT index) among patients on the CMS protocols was 31% less than among controls, and the overall DMFT increment among CMS patients was 21% less than in controls 15. A cost‐effectiveness evaluation, from the perspective of the dentist provider, was conducted 16. This showed substantial effects (numbers of avoided DMFT increments in CMS patients) for modest additional costs above those incurred by control patients. Sensitivity analyses demonstrated that cost‐effectiveness was improved for high‐risk patients and for those treated by hygienists.

The success of the clinical trial prompted preparation for a continuation of the program for the threefold purpose of determining whether the new mode of practice would be sustained post‐trial in the absence of monitoring visits (a real‐life situation as opposed to conditions operating under a controlled clinical trial); to assess cost‐effectiveness over a longer term; and to conduct a qualitative evaluation 15.

The purpose of this article was to report on the potential legacy of the trial, that is, whether or not the beneficial effect was sustained, at two and 4 years post‐trial, among patients attending CMS practices.

## Methods

### Practice and patient recruitment

**Figure 1:**
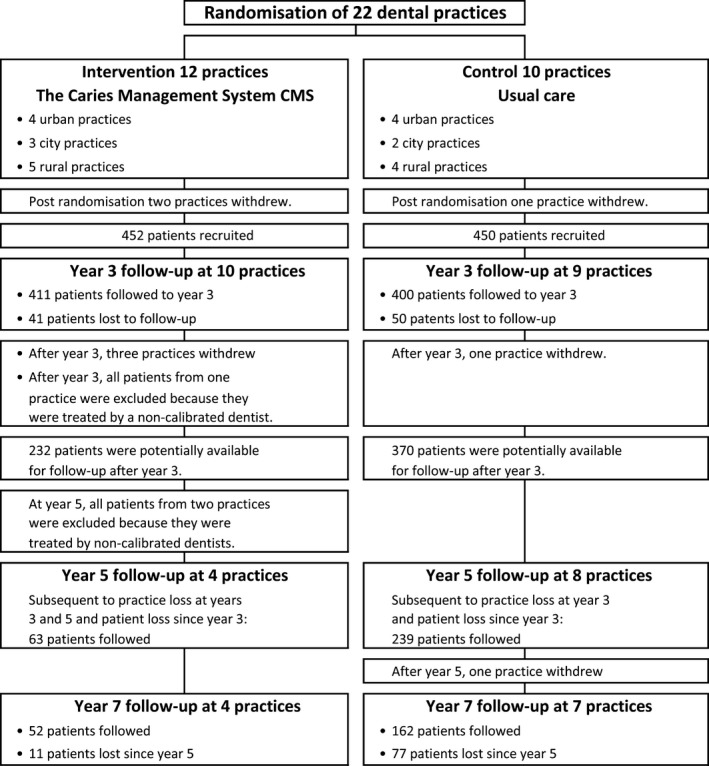
Study flow diagram.

Twenty‐two practices were recruited and randomized to participate in the controlled clinical trial; 12 to intervention and 10 to control. Immediately postrandomization, three practices withdrew (Fig. 1) 13. Typically, the first 50 consecutive patients who consented to participate were enrolled. At the control practices, caries management following usual practice would continue to be delivered. Practices were located in city, urban, and rural locations in both fluoridated and nonfluoridated communities.

### Practice and patient loss

**Figure 2:**
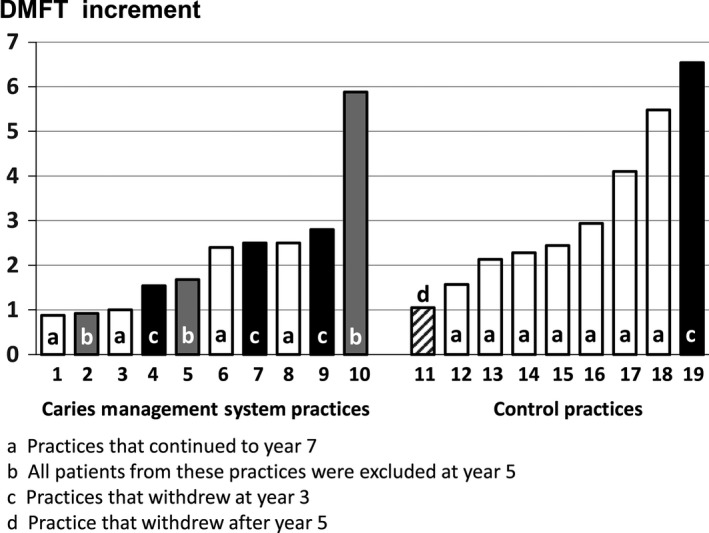
Year 3 distribution of patient mean DMFT increments by intervention and practice participation postclinical trial.

Immediately post‐trial, three CMS practices withdrew (retirement of the calibrated dentist, practice sale, and loss of interest (Figs 1 and 2). In addition, one control practice was lost due to practice sale. At year 5 (2 years post‐trial), all patients attending three CMS practices were excluded because they were treated by noncalibrated dentists. After year 5, one control dentist ceased practicing.

### Potential bias

As a consequence of practice and patient loss, a potential to bias the post‐trial follow‐up emerged (Table 1). At baseline, the mean DMFT scores of the CMS and control patients were equal at 8.5 and 8.4, respectively (Table 2). However, for those in both study arms continuing to years 5 and 7, the baseline scores were in balance (CMS 12.2 and control 12.6); the lost patients had lower caries experience.

**Table 1:** Frequency distribution of practices by location and fluoridation status

Follow‐up period	Intervention	City	Urban	Rural	Total^a^	Fluoridated	
						Yes	No
Baseline to year 3	CMS^b^	2	3	5	10	8	2
	Control	2	3	4	9	7	2
Years 4 and 5	CMS	1	2	1	4	4	0
	Control	2	3	3	8	7	1
Years 4–7	CMS	1	2	1	4	4	0
	Control	1	3	3	7	6	1

a Twenty‐two practices were randomized at baseline but three withdrew prior to patient recruitment.

b Caries Management System.

**Table 2:** Caries Management System arm and Control arm mean baseline DMFT balance by cohort

Cohort	Practice numbers	Total patients	Caries Management System			Control			Probability^a^
			*n*	Mean	SD	*n*	Mean	SD	
All patients on enrollment	19	902	452	8.5	8.2	450	8.4	8.6	0.82
All patients who continued to year 3	19	811	411	9.2	7.7	400	12.0	8.4	<0.0001
All patients who continued to year 5	12	302	63	12.1	7.4	239	11.7	8.1	0.74
All patients who continued to year 7	11	214	52	12.2	7.5	162	12.6	7.9	0.74

a Wilcoxon test comparing CMS against Control.

### Patient recall and data collection

Dentists were requested to recall patients for monitoring and related treatment prior to the dates set for data collection at two and 4 years of post‐trial (five and 7 years postbaseline), respectively. If, at year 7, patients had not attended recall appointments or received further treatment after year 3, they were deemed to be lost to follow‐up at year 5. Data were not collected in relation to patient behavior change. Only treatment‐related data were taken directly from patient files at each practice by the researchers and entered electronically for later analysis. As such, the researchers were not blinded to either practice allocation or clinical outcome. For the purposes of the post‐trial analysis, all interventions reported during years 4 and 5 and during years 6 and 7 were registered as occurring at year 5 and year 7, respectively (last observation carried forward, LOCF).

### Determination of DMFT increment

Tooth surface data pertaining to each patient were inspected and both DMFS and DMFT increments were calculated. During the 3‐year clinical trial, the D component of the increment score was monitored and recorded separately. An approximal tooth surface was registered as D if bitewing radiolucencies were disclosed in enamel or dentine during monitoring visits. Both D incidence and progression were reported at years 2 and 3 but since monitoring visits ceased after year 3, the D component of the DMFT scores could not be reported. During the post‐trial period, it was assumed that any replacement fillings and first time fillings were consequent to decay and, therefore, the D increment was reflected in F, where the DMFT increment was estimated as M+F during years 4–7. During the clinical trial, care was taken where possible to register replacement fillings as decay‐related events only if they were consequent to decay, that is, broken fillings or fillings that were replaced for other reasons were not registered as F increments at the patient level 14. However, in the post‐trial analyses, F was calculated as the sum of all restoration‐related events: first time fillings, crowns, replacement fillings including replacement crowns, root fillings, bridge units, denture units, and implants. This post‐trial policy was justified in that the long‐term economic evaluation should capture the impact of the totality of restorative care needed consequent to treatment emanating from an original caries diagnosis.

### Data analysis

The patient outcome measure was the cumulative increment in the DMFT index score and the practice outcome measures included the practice‐mean and practice‐median increments of patient DMFT index scores. Comparisons of baseline mean DMFT estimates between CMS and control patients were assessed using the Wilcoxon rank‐sum test. In covariable analysis (patient‐level unit of analysis), as the patients were clustered by practices, mean DMFT increments were determined through multilevel modeling analysis. Two significant risk factors, in addition to intervention, were jointly associated with DMFT increments: age on enrollment and baseline DMFT. In addition, practice‐mean DMFT increments (practice‐level unit of analysis) and practice‐median DMFT increments (also practice level) were determined through general linear modeling analysis of covariance.

A multiple variable logistic regression analysis of caries risk status was conducted in which risk was dichotomized as high risk (those whose DMFT increments increased by more than one per year) verses both low and medium risk combined (those with increments of one or fewer per year). Odds ratios were adjusted for age on enrollment, baseline DMFT, fluoridation status, practice location, and intervention, which were the four significant risk factors found based on our data.

All analyses were conducted using sas (version 9.3) software (SAS Institute Inc., Cary, NC, USA).

### Ethics

Approval was obtained from the University of Sydney Human Research Ethics Committee to monitor the post‐trial effect, and then dentists and patients were invited to give their informed consent to continue their participation.

## Results

Two years post‐trial, 509 patients were lost to follow‐up leaving 302 on protocol or under usual care. After 4 years, the effect of the post‐trial legacy was assessed in relation to only 214 remaining patients −52 attending four CMS practices (12% of the baseline number) and 162 patients attending seven control practices (36% of the baseline number) (Fig. 1).

### Patient‐level DMFT increments

The year 5 (2‐years post‐trial) mean DMFT increments for CMS and control patients (adjusted for baseline DMFT and baseline age) were 1.21 and 1.53 (*P* = 0.32), respectively (Table 3). During the second 2‐year period post‐trial (years 6–7), the respective mean increments of 1.23 and 1.80 differed significantly (*P* < 0.05). The overall 4‐year post‐trial DMFT increments (years 4–7) for CMS and control patients were 2.44 and 3.39 (*P* < 0.01), respectively; a difference equivalent to 28%. Finally, the effect of the CMS protocols on patients as measured from the clinical trial baseline to the end of the post‐trial follow‐up (year 7) was a DMFT increment of 6.13 which was 29% less than the increment of 8.66 for control patients (*P* < 0.0001).

**Table 3:** Post‐trial patient‐level and practice‐level DMFT^a^ increments and related statistics by treatment period and intervention – Caries Management System (CMS) versus Control – LOCF^b^

Treatment period	Intervention	Practice numbers	Patient‐level statistics						Probability^d^	% Difference^d^
			*N*	Median	Mode	Max	Adjusted mean^c^	SE^c^		
First post‐trial period (Years 4–5)	CMS	4	63	0	0	10	1.21	0.29	0.3234	20.9
	Control	8	239	1	0	10	1.53	0.18		
Second post‐trial period (Years 6–7)	CMS	4	52	1	0	5	1.23	0.24	**0.0385**	31.7
	Control	7	162	1	0	24	1.80	0.15		
Post‐trial period (Years 4–7)	CMS	4	52	1	0	10	2.44	0.31	**0.0075**	28.0
	Control	7	162	2	0	27	3.39	0.20		
Baseline – year 3	CMS	6	63	1	0	11	3.99	0.55	**0.0064**	37.7
	Control	8	239	2	0	22	5.44	0.39		
Baseline – year 5	CMS	4	63	2	0	19	4.95	0.55	**0.0012**	25.8
	Control	8	239	3	0	27	6.67	0.39		
Baseline – year 7	CMS	4	52	3.5	0	19	6.13	0.58	**<0.0001**	29.2
	Control	7	162	5	3	32	8.66	0.41		

			Practice mean	Practice‐level statistics					Probability^d^	% Difference^d^
				SE	Probability^d^	% Difference^d^	Practice median	IQR		
First post‐trial period (Years 4–5)	CMS	4	1.07	0.27	0.4111	18.3	0.50	1.00	0.7248	19.3
	Control	8	1.31	0.21			0.62	1.75		
Second post‐trial period (Years 6–7)	CMS	4	1.08	0.30	0.0821	35.3	0.75	1.00	0.9215	−5.6
	Control	7	1.67	0.25			0.71	1.75		
Post‐trial period (Years 4–7)	CMS	4	2.16	0.43	0.0555	30.3	1.25	0.38	**0.0394**	47.0
	Control	7	3.10	0.36			2.36	1.00		
Baseline – year 3	CMS	6	1.55	0.40	**0.0130**	43.2	0.10	1.25	0.3384	93.3
	Control	8	2.73	0.36			1.50	2.25		
Baseline – year 5	CMS	4	3.33	0.79	0.1872	25.6	2.50	2.38	0.3666	23.1
	Control	8	4.48	0.63			3.25	3.00		
Baseline – year 7	CMS	4	4.38	0.85	**0.0290**	33.1	2.87	1.75	**0.0056**	46.5
	Control	7	6.55	0.71			5.36	4.38		

Bold indicates significance (*P* < 0.05).

a Calculated as M+F where F = sum of all (i) first time fillings, (ii) crowns, (iii) repeat fillings including repeat crowns, (iv) root fillings, (v) bridge units, (vi) denture units, and (vii) implants.

b Last observation carried forward.

c Mean values are adjusted for baseline DMFT and baseline age. SE of adjusted mean.

d Relate to CMS versus Control differences.

### Practice‐level estimates of DMFT increments

The caries experience of patients, expressed as both practice‐mean and practice‐median DMFT increments, are also reported in Table 3. During the first 2‐year post‐trial period (years 4–5), the difference between the practice‐mean increments across the practice types was not significant (*P* = 0.41). Also, the difference during the second 2‐year period (years 6–7) was not significant (*P* = 0.08). Over the entire post‐trial period, the CMS practice‐mean DMFT increment of 2.16 was 30% lower than the control mean increment of 3.10; however, the difference was at the margin of statistical significance only (*P* = 0.0555). Over the 7‐year period from baseline, the difference at year 7 of 33% related to CMS and control mean increments of 4.38 and 6.55, respectively (*P* = 0.029).

The practice‐median DMFT increments were lower than the practice‐mean increments (Table 3). During each of the 2‐year post‐trial periods, the practice‐median increments were not significantly different across practice types. On the other hand, there was a significant 47% difference between the median increments during the 4‐year post‐trial period (years 4–7) at which point the respective CMS and control practice medians were 1.25 and 2.36 (*P* = 0.039). During the periods from baseline to years 3, 5, and 7, a difference between the practice‐median increments was significant in the case of the baseline to year 7 median only; the respective CMS and control median estimates being 2.87 and 5.36, a 47% difference (*P* < 0.006).

### Analysis of risks factors

Minimally elevated odds (reported as adjusted odds ratios in Table 4) of being high risk were associated with baseline DMFT (OR = 1.17). Patients attending the CMS practices had lower odds of being high risk than those attending control practices (OR = 0.23, 95% CI = 0.06, 0.88). Neither age on enrollment nor current exposure to fluoridated water was significantly associated with reduced odds of being high risk.

**Table 4:** Logistic regression analysis of high caries risk status.^a^ Odds ratios adjusted for risk factors

Risk factor	OR	CI95
Age on enrollment	1.022	0.998, 1.046
Baseline DMFT	1.168	**1.099, 1.242**
Fluoride history^b^	0.154	0.018, 1.307
Intervention (CMS)^c^	0.228	**0.059, 0.880**

Bold indicates significance (*P* < 0.05).

a Dichotomized as high risk versus low or medium risk.

b Current exposure to water fluoridation. Comparator reference = Noncurrent exposure.

c Comparator reference = Control (usual care).

## Discussion

This study has demonstrated that during the post‐trial period, the reduced need for restorative care was sustained among patients attending dental practices that implemented the CMS nonsurgical preventive treatment protocols for caries control. Mean patient needs for restorative treatment during this period were 28% fewer than those under usual care at control practices. Further, during the 7‐year postbaseline period, the mean need for such treatment among CMS patients was 29% fewer than that of control patients. At the practice level, the mean number of restorations placed by CMS dentists was 30% fewer (at the margin of statistical significance only) than that of control dentists during the post‐trial period, and 33% fewer during the 7‐year period from baseline. The CMS practice‐median number of restorative interventions during both the post‐trial and 7‐year periods was substantially fewer (47%) than at control practices. These results align with those of Featherstone et al. 17 who found that systematic monitoring and professional applications of fluoride and other anticaries measures reduced caries incidence and need for restorative intervention among caries active adult patients.

The patient‐level analysis found most of the differences in the treatment effects discussed in the previous paragraph significant at level 0.05, especially all those treatment effects of main interest, whereas the practice‐level analysis failed to reach significance in most of the differences in the treatment effects. It is noted, however, that patient‐level significance should imply that practice level is also significant. The main reason for the larger *P*‐values in the practice‐level analysis is purely statistical; there were only four data points in the CMS arm and seven data points in the control arm, which makes the sample size too small to detect a significant effect. Also, it would be more appropriate to develop a statistical method to incorporate the estimated variances of the practice‐level means into the analysis, which could not be fulfilled using standard procedures such as in SAS. Hence, we kept our analysis as simple as possible, hoping that the reader would focus on the more reliable patient‐level analysis, for which the sample size was much larger.

As far as we are aware, there are no other practice‐based randomized controlled trials of preventive protocols with which to compare our results. Nevertheless, it is realistic to speculate that similar results might be realized if preventive treatment was to be adopted as standard practice for caries control. The fact that this study was conducted in the real‐life setting of private dental practice lends additional strength to any generalization of the findings.

The main limitation to the interpretation of this study outcome is severe patient attrition. Initially, this study was planned as a 3‐year clinical trial and powered accordingly with provision for dropouts (3). When the opportunity arose to continue the study, we were left with the numbers in‐hand who continued to years 5 and 7. In real‐life dentistry, both patients and dentists come and go. In Australia, population mobility, as assessed in the 2008 household survey, was such that 43% had moved house in the last 5 years 18. Attrition was due to a combination of loss of patients within continuing practices, practice withdrawals, and practice exclusions. The lost practices in both arms of the study were distributed across the full range of the year 3 practice‐based mean DMFT increments (Fig. 2), and hence the practice profiles in both study arms were preserved. Three CMS practices were excluded due to patient treatment by non‐calibrated dentists. At these practices, a high incidence of restoration of previously nonrestored teeth was found and it is likely that many of the restored lesions were probably arrested lesions. This regrettable outcome is not surprising given the long‐standing caries treatment philosophy favoring early restorative intervention. As the focus of the post‐trial follow‐up was to determine whether continuing patients on protocol would sustain reduced caries risk, these practices were, therefore, excluded. While the lost patients had, on average, lower baseline DMFT scores than those continuing, baseline DMFT equality across both study arms was maintained. The dropout due to population mobility, classed as missing completely at random, does not introduce significant bias theoretically. Two of the three lost practices were also missing completely at random. The remaining lost CMS practice was due to loss of interest and may (not necessarily) introduce over‐optimistic bias. Nevertheless, severe attrition demands that caution be exercised when interpreting the study outcome.

It is presumed that the dentists who elected to join the study and were randomized to CMS or control, were preventively oriented and, therefore, constitute a biased sample. Less preventively oriented dentists might, when erring on the side of ‘safety’, adopt an earlier rather than a later stage of lesion development as their threshold for surgical intervention.

We were unable to monitor caries lesion incidence during the post‐trial follow‐up, hence our assumption that F increments to DMFT scores reflected D incidence may not be the case. It is possible that the observed lower mean DMFT increments in CMS practices during the RCT and the post‐trial period were due entirely to a change in diagnostic/treatment threshold of the participating dentists and without any effect due to caries preventive behavior changes by the patients themselves. However, the effect favoring CMS patients was more likely due to a combination of change in diagnostic/treatment threshold and lesion arrest due to intensive fluoride therapy, and decreased lesion incidence due to the preventive approach of the CMS. This outcome could also be reached without the benefits of home preventive activities by patients. Similarly, the observed reduction in caries risk may be a misinterpretation wherein caries risk itself may not have changed; rather the decrease in restorative need being due to changed practice by CMS dentists who refrained from restoring early lesions. This alone leads to more patients being classified as low risk.

While the CMS protocols have a joint focus on behavioral and clinical management, the control of dental plaque and sugar exposure were not monitored by the researchers during the MPP. Hence, the extent to which control of these factors affected the clinical outcome is not known and represents a weakness in the study. As it is desirable that unnecessary restorative care is avoided, this result is best achieved through the joint preventive efforts of both patient and dentist to reduce caries risk and need for restorative intervention. Nevertheless, best clinical practice alone can go a long way to eliminate need for restorative intervention.

It was believed that caries could be controlled via the invasive practice of restorative treatment of caries lesions. Griffin et al. 19 exposed the inherent flaw in this approach in their review of epidemiologic studies conducted in four industrialized countries. They revealed that the annual coronal caries increment was 0.86 (CI95 0.66–1.07) surfaces per year. Under the practice of erring on the side of safety as a justification for early surgical intervention, the risk of over‐treatment is serious. Data from Japan indicate that 74% of dentists would intervene surgically on enamel lesions in high‐risk patients and that 47% would do so in low risk patients 20. Similar intentions were expressed by dentists in Victoria, Australia 21. In Australia, data from the most recent national survey of randomly selected dentists indicate that on any 1 day, equal numbers of enamel and dentine lesions are restored 22. However, Brown et al. 23 showed that in a population, described as ‘representing a typical, contemporary, caries active adult population with fluoride exposure’, half of noncavitated lesions reversed during a 33 month period, around one quarter remained stable, around one‐fifth oscillated between progressing and regressing, and only 8.3% progressed to cavitation. These results highlight the potential for lesion monitoring and active arrestment/preventive strategies as the new paradigm for caries control. Anusavice noted that if modern management is to be successful, caries risk must be monitored and lesions under treatment must also be monitored to track their activity and to adjust treatment when indicated 24.

A major concern of the restorative approach, in addition to overtreatment, is the concomitant loss of tooth structural integrity. The need to preserve dental tissues should be the guiding principle in future caries management 25. Provided that patients attend regularly for preventive treatment according to risk‐specific monitoring schedules, their need for invasive restorative treatment with its attendant life‐long maintenance requirements is unnecessary and avoidable 7, 26. Gilbert et al. 27 have reported that dentists who belong to practice‐based research networks, and who are following evidence‐based protocols become, in time, less invasive in their treatment approach. Recall for monitoring and ongoing preventive treatment ensures that most new incident lesions should be identified, arrested, and remineralized. However, research outcomes alone from the studies reported here do not ensure that they will be translated into practice. Bader identified three critical areas that govern dental practice: interaction between dentists’ characteristics and biases; the environment of dental practice and its effects on treatment decisions; and dentists incorporation of the values of their patients into their treatment decisions 28. Our qualitative evaluation of the MPP indicates that dentists value the benefits of noninvasive practice including that of practice building and patients highly value the main benefit of a reduced need for restorative treatment 29, 30. Further, the economic evaluation shows that preventive practice is economically sustainable 15, 16.

It is concluded that in practices where adherence to the CMS protocols was maintained during the 4‐year post‐trial follow‐up period, patients experienced lower needs for restorative treatment. The outcome of this clinical trial and its sustained legacy should stimulate dental schools and the profession‐at‐large to reshape their approach to caries management. As restorative care is not appropriate for non‐cavitated lesions, the persistence of a restorative approach to caries control results in unnecessary invasive procedures, and brings into question the ethics of continuing this practice.
